# Sex Differences in Spatial Memory: Comparison of Three Tasks Using the Same Virtual Context

**DOI:** 10.3390/brainsci11060757

**Published:** 2021-06-07

**Authors:** Laura Tascón, Carmen Di Cicco, Laura Piccardi, Massimiliano Palmiero, Alessia Bocchi, José Manuel Cimadevilla

**Affiliations:** 1 Department of Psychology, University of Cordoba, 14071 Cordoba, Spain; 2 Department of Biotechnological and Applied Clinical Sciences, University of L’Aquila, 67100 L’Aquila, Italy; carmendicicco.psy@gmail.com (C.D.C.); massimiliano.palmiero@univaq.it (M.P.); alessia.bocchi@gmail.com (A.B.); 3 Department of Psychology, Sapienza University of Rome, 00185 Rome, Italy; laura.piccardi@uniroma1.it; 4 Cognitive and Motor Rehabilitation and Neuroimaging Unit, IRCCS Fondazione Santa Lucia, 00179 Rome, Italy; 5 Department of Psychology, University of Almeria, 04120 Almeria, Spain; jcimadev@ual.es; 6 Health Research Center, University of Almeria, 04120 Almeria, Spain

**Keywords:** spatial navigation, spatial orientation, sex differences, dimorphism, virtual reality, spatial tests, cognitive load

## Abstract

Spatial memory has been studied through different instruments and tools with different modalities of administration. The cognitive load varies depending on the measure used and it should be taken into account to correctly interpret results. The aim of this research was to analyze how men and women perform three different spatial memory tasks with the same spatial context but with different cognitive demands. A total of 287 undergraduate students from the University of Almeria (Spain) and the University of L’Aquila (Italy) participated in the study. They were divided into three groups balanced by sex according to the spatial memory test they performed: the Walking Space Boxes Room Task (WSBRT), the Almeria Spatial Memory Recognition Test (ASMRT) and the Non-Walking Space Boxes Room Task (NWSBRT). Time spent and number of errors/correct answers were registered for analysis. In relation to the WSBRT and the ASMRT, men were faster and reached the optimal level of performance before women. In the three tests, familiarity with the spatial context helped to reduce the number of errors, regardless of the level of difficulty. In conclusion, sex differences were determined by the familiarity with the spatial context, the difficulty level of the task, the active or passive role of the participant and the amount of visual information provided in each screen shot.

## 1. Introduction

Spatial orientation is the ability to successfully reach certain places or objectives through space [1]. Memory has an important role during spatial orientation since it is essential to recall or plan a route to reach a place [2]. This kind of memory is called topographic memory and involves not only visuospatial information, but also vestibular and proprioceptive inputs relative to whole-body movements, as well as a continuous updating of the person’s perspective during his movements in the environment (e.g., [3,4]).

Topographical memory is largely supported by object location memory, which contributes to maintaining a coherent and meaningful representation of the visual world, as well as generating directional information [5,6,7].

In the last few decades, the development of new technologies has simplified the assessment of spatial memory in humans [8,9,10,11,12] and has made possible the study of topographic memory, which implies large spaces in the real environment that can be reproduced to scale in the virtual environment while also controlling all the elements of the environment itself. This has allowed the development of a high number of studies belonging to a wide range of fields of knowledge such as neuroscience, developmental psychology, geography or security [13,14,15].

In line with the foregoing, virtual-reality-based tasks offer multiple options for the study of spatial memory: they allow the usage of different sceneries and provide the possibility of modifying the amount and location of landmarks [16,17,18,19]. According to this, two different sorts of virtual spatial tasks can be found when considering the role of the participant: active and passive tasks. In active tasks, participants can freely explore the experimental scenery with the help of a peripheral (a joystick, a mouse, a keyboard, etc.) [8,9,20,21]. All participants have a unique and personal experience since they can take several paths or spend different amounts of time interacting with the stimuli. Conversely, in passive tasks, participants are mere observers. All participants have the same navigational experience [17,22,23,24]. Moreover, sometimes the context is shown from different static points of view [16,22,25,26]. Successful orientation demands combination of spatial information available from different viewpoints.

Moreover, all these spatial tasks can be solved using different spatial strategies. On the one hand, egocentric strategies take into account body-centered representations. The target or goal is represented in relation to the observer. On the other hand, allocentric strategies are based on the information provided by the environment and depend on the flexible representation between the goal and stimuli available in the context [27]. Allocentric strategies are related to the integrity of the medial temporal lobe [9,20,28,29].

Systems underpinning both types of strategies work in parallel [27]; thus, the experimental isolation of both spatial representations is particularly challenging [30]. Even so, certain experimental conditions could cause participants to use one strategy or another with a higher probability [27,29,31]. Recognizing spatial locations from different viewpoints has been reported to depend on the medial temporal lobe and to demand the use of allocentric reference frames. The use of egocentric strategies is also partially impeded when the starting position is moved between trials in an active task. In both situations, development of a flexible spatial relationship between the spatial cues available in the context is the best option for an accurate orientation.

Furthermore, there are a great number of factors related to the task design that must be considered to correctly interpret the spatial behavior, such as the environment [32]; the time available to memorize the scenery [31,33]; the level of difficulty [34,35,36,37]; the visual perspective taken of the scenery or alignment effect [33,38,39,40]; and other individual variables, such as sex [41,42,43,44,45,46,47,48], age [47,48], cognitive styles [19,44,49], familiarity or degree of knowledge of the spatial context [50,51] and other psychological factors [32,35,42,43] that can modulate the final spatial behavior.

As mentioned above, sex can interact with the preference of spatial strategies. Indeed, in spatial orientation as well as in topographic memory there are strong evidences that men and women differ (for a review see: [52,53]). Thus, men tend to use a “survey” strategy, based on a map-like representation of landmarks and the spatial relationships between them. In addition, they organize the spatial information allocentrically. On the contrary, women prefer a “route” strategy, where the sequence of appearance of landmarks is essential [36]. Spatial strategies used by men are more adaptive in allocentric spatial tasks, creating cognitive maps and recognizing spatial environments independently of the point of view [35,54]. Taking this into account, women could show more difficulties in passive tests, since it is impossible to develop tracks or routes and accurate solutions involve the use of allocentric strategies: combination of spatial information taken from different locations independent of the position of the observer.

Furthermore, sex dimorphism also appears under high cognitive demands, i.e., in conditions when the task requires high visuospatial working memory load besides the use of allocentric strategies [33,35]. In addition to this, the use of different strategies in solving a navigational task was also supported by the involvement of different neural correlates in men and women. In fact, in a study by Grön and colleagues (2000) [55], it was observed that women tend to use more prefrontal areas, suggesting a large load on the visuospatial working memory, in contrast to men, who solve the same task using the navigational network that would be naturally involved.

At last, it is important to highlight that familiarization with the environment can cause the sexual differences described above to disappear. Nori and colleagues (2018) [31] and Piccardi et al. (2011) [33] observed how sex differences decreased or disappeared when participants were allowed to take all the time they needed to learn the spatial information. Moreover, a good knowledge of the environment allows better performance of highly demanding spatial tasks [51].

As already mentioned above, spatial memory has been studied through a large number of different instruments and tools that have different designs and characteristics and, therefore, different cognitive demands. This is why all these factors mentioned above have to be taken into account in order to correctly interpret the results. With this in mind, the aim of this research was to analyze the performance of men and women in three spatial memory tasks under different conditions: different difficulty levels and active or passive role of participants. Tasks were developed in the same virtual environment but demands were changed: the Walking Space Boxes Room Task (WSBRT) is an active task where participants can freely move and explore the virtual room to find rewarded positions; the Non-Walking Space Boxes Room Task (NWSBRT) is a passive task where participants can see the virtual context from any of the four walls to find the rewarded positions. Finally, the Almeria Spatial Memory Recognition Task (ASMRT) is a passive task where participants have to memorize rewards’ positions and thereafter have to recognize correct locations from several viewpoints. To our knowledge, this was the first study that investigated gender differences through the application of three different tests with the same virtual environment. Each test has slightly different demands, which allows a more detailed analysis of the factors that determine sex dimorphism.

In line with the literature, we hypothesized that sex differences will appear under specific demands: high visuospatial working memory load and passive tests. Familiarity with the environment will have a positive impact on the execution of all tests, especially in women.

## 2. Materials and Methods

### 2.1. Participants

The sample was composed of 287 undergraduate students from the University of Almeria (Spain) and the University of L’Aquila (Italy). They were randomly divided into three groups: the Walking Space Boxes Room Task (WSBRT), the Almeria Spatial Memory Recognition Test (ASMRT) and the Non-Walking Space Boxes Room Task (NWSBRT) (see Table 1 for demographic details). Participants reported in a questionnaire that they did not have any psychological or neurological disorder that could affect the performance of the tasks. Previous studies in Italian and Spanish participants did not disclose nationality-based differences (unpublished results).

The study was conducted under the Code of Ethics of the World Medical Association (Declaration of Helsinki) for experiments involving humans.

### 2.2. Apparatus

The three tasks were presented on a Hewlett Packard 2600-MHz notebook equipped with 3 GB of RAM and a 15.4 Thin Film Transistor (TFT) color screen (1920 × 1200 pixels). A joystick, a keyboard and a mouse were used for the WSBRT, the ASMRT and the NWSBRT, respectively.

### 2.3. Procedure

Both Spanish and Italian researchers applied the three tests under the same experimental conditions: participants were tested individually by one researcher inside a quiet room. Participants received written instructions for the task. Each participant performed one of the three spatial tests that are explained in detail below:

#### 2.3.1. The Walking Space Boxes Room Task (WSBRT)

**Figure 1:**
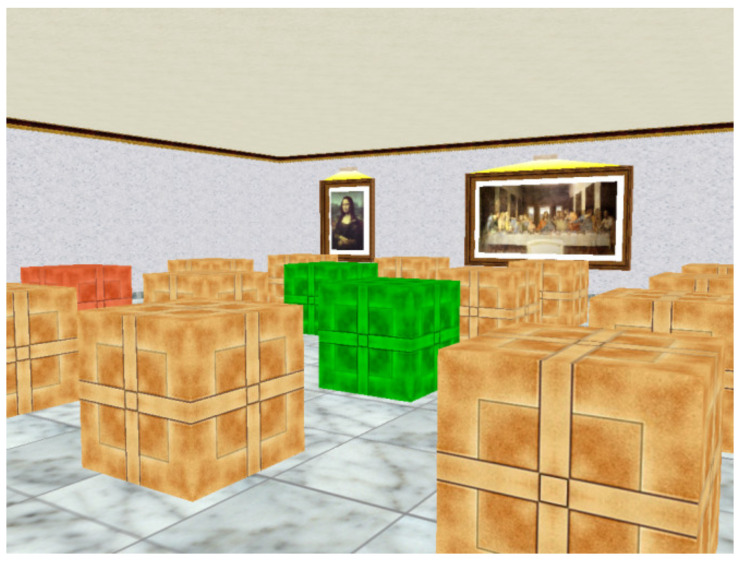
View of the WSBRT.

The WSBRT, also known as the Boxes Room Task [34], is a spatial memory test developed to evaluate place learning. It is based on the hole-board maze used for rodents. In this case, participants had an active role and they used a joystick to freely move within the scenery. They had a first-person view of a square room that contains several stimuli such as pictures, a window and a door (see Figure 1). A total of 16 brown boxes ordered in four rows were on the floor and some of them were rewarded. Their objective was to discover and remember the position of the rewarded boxes. Participants could move around and select those boxes by pressing the bottom of the joystick. At the beginning, they had to locate the rewarded boxes by trial and error and then recall them later. If a rewarded box was selected, it changed to a green color and a pleasant melody sounded. Conversely, if it was a wrong box, it changed to a red color and an unpleasant melody sounded. Participants had to memorize the position of the green boxes, which remained in the same position throughout the whole experiment. Once all the green boxes had been found or after 150 s, a new trial started. The starting position, from any of the four room walls, changed in every trial, avoiding egocentric solutions. Three levels of difficulty were applied, where participants had to memorize the location of 3, 5 or 7 green boxes. All participants performed the three difficulty levels in an ascending order. Each difficulty condition was composed of 10 trials. Since the first trial participants did not have any information about the location of the correct boxes, they opened them randomly. Both number of errors (number of non-rewarded boxes opened) and latencies (time spent to find all the rewarded boxes) were registered.

#### 2.3.2. The Non-Walking Space Boxes Room Task (NWSBRT)

**Figure 2:**
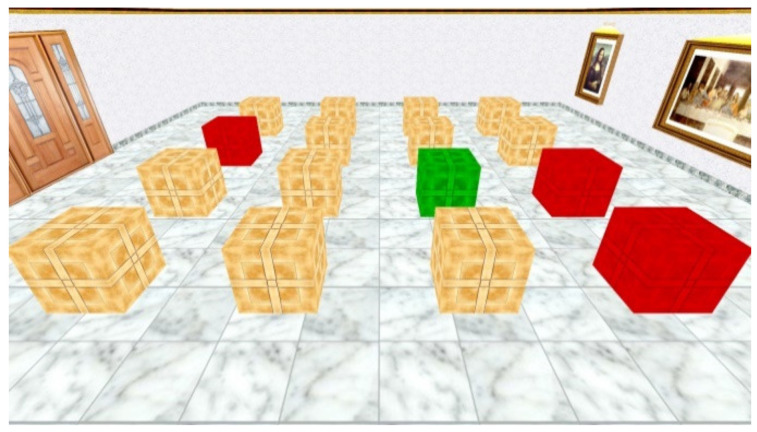
View of the virtual room in the NWSBRT. Note that the context is the same as in the WSBRT and ASMRT tasks.

The scenery of the NWSBRT [26] was the same as in the WSBRT; however, participants could not walk inside the room (see Figure 2). They had a survey perspective of the same room and could see the entire environment at a single glance, except for one wall. In the room, the same landmarks and the same 16 boxes as in the WSBRT appeared. The objective of the task was also to find the rewarded boxes. In this case, participants could open the boxes by clicking on them with the mouse. Rewarded boxes turned to green whereas wrong boxes turned to red. Each trial finished when participants found all the green boxes or after 60 s had elapsed. The point of view changed randomly to a different wall in each new trial and the location of the rewarded boxes was the same during the whole experiment. Participants had to find 3, 5 or 7 rewarded boxes, depending on the difficulty level. All of them performed the three levels of difficulty in an ascending order. Each difficulty condition was composed of 10 trials. Accuracy (number of non-rewarded boxes opened) and latencies (time spent to find all the rewarded boxes) were recorded in each trial. Before starting, participants received written instructions.

#### 2.3.3. The Almeria Spatial Memory Recognition Test (ASMRT)

**Figure 3:**
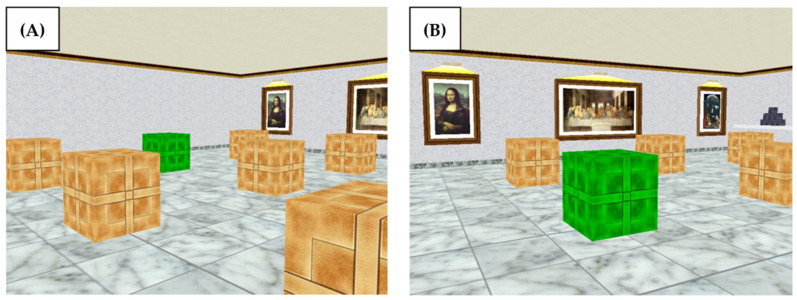
ASMRT: Example of a memorizing image (**A**) and a recognition image (**B**). The answer is correct and participants should press the “yes” key because the green box in the recognition image occupies the same location as in the memorizing image.

The ASMRT is a spatial memory task based on a recognition paradigm [25]. The test is composed of four trials, each one divided into two parts: a memorization phase and a recognition phase. In the first phase, participants were shown an image of a virtual room that contains 9 boxes with 1, 2 or 3 of them in green color, according to the difficulty level. Participants had to memorize the position of the green boxes. In the recognition phase, ten images of the same room were shown from a different point of view, containing one green box. Participants had to decide if the green box of the recognition image occupied the same location as any of the green boxes in the memorizing image (see Figure 3). There was no time limit for memorization. The recognition phase started when participants pressed the space key after memorizing. A total of 10 recognition images were displayed one by one. For each one, participants had to decide by pressing a “yes” key or a “no” key. Five of the pictures shown were correct. Three levels of difficulty were applied for each participant in an ascending order. They had to memorize a total of 1, 2 or 3 locations. Accuracy (number of correct answers) and reaction times were recorded. Instructions were presented on the screen of the computer. See Table 2 for a schedule of the three tests.

For the subsequent interpretation of the results, it is important to define the concept of familiarity. In this study, familiarity is the grade of knowledge of the room, the elements inside and the position of the boxes within it. This knowledge increases as participants complete each trial and each level of difficulty. It is well known that, as participants are more familiarized with the environment, they take more advantage to find the rewarded boxes [31,51]. On the other hand, the asymptotic level indicates when participants have reached their optimal level of performance. Therefore, in this study we used this data to determine when participants might be more familiarized with the environment.

### 2.4. Statistical Analyses

As dependent factors, mean number of errors and latencies/trial in the WSBRT and NWSBRT were analyzed using a three-way mixed ANOVA (Sex × Difficulty × Trial) with Sex as a between factor and Difficulty and Trial as within factors. The first trial was discarded since the targets’ positions were unknown and, accordingly, participants opened boxes randomly. Sex, Difficulty and Trial were the independent variables.

Regarding the ASMRT, number of correct answers and reaction times were analyzed with a two-way ANOVA (Sex × Difficulty), with Sex as a between factor and Difficulty as a within factor. Again, Sex and Difficulty were the independent factors.

Newman–Keuls test was applied for all post hoc analyses. Differences were considered statistically significant for *p* < 0.05. STATISTICA 10 was used for running all analyses.

Difficulty was established by the number of locations to memorize while familiarity was determined by the asymptotic level. Due to the configuration of the task, asymptotic levels were not described in the ASMRT.

## 3. Results

### 3.1. WSBRT

#### 3.1.1. Number of Errors

ANOVA (Difficulty x Trial x Sex) revealed significant main effect of Sex F(1, 87) = 9.79, *p* = 0.002, η_2_ = 0.101; Difficulty F(2, 174) = 25.63, *p* < 0.000, η_2_ = 0.227; and Trial F(8, 696) = 92.96, *p* < 0.000, η_2_ = 0.516. Two significant interactions appeared: Sex × Trial F(8, 696)= 3.62, *p*< 0.001, η_2_ = 0.039 and Difficulty × Trial F(16, 1392) = 13.11, *p* < 0.000, η_2_ = 0.131.

**Figure 4:**
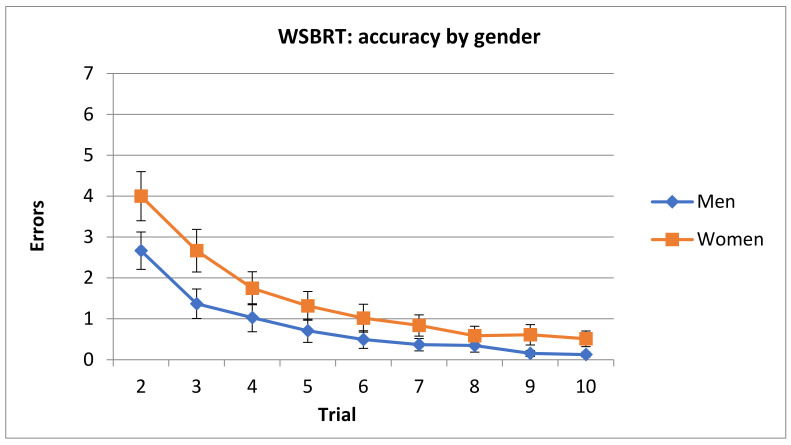
Mean number of errors committed in the WSBRT divided by trials. Note that trial 1 was removed from analysis since performance was at random. (Mean ± SEM).

Post hoc analysis of the interaction term Sex x Trial revealed that men reached the asymptotic level in trial 5, whereas women did it in trial 6. In addition, number of errors in trials 2 and 3 was smaller in men (see Figure 4).

In relation to post hoc analyses of the interaction term Difficulty x Trial, the asymptotic level was reached in trial 6 in 3 locations’ condition and in trial 4 in conditions with 5 and 7 locations.

**Figure 5:**
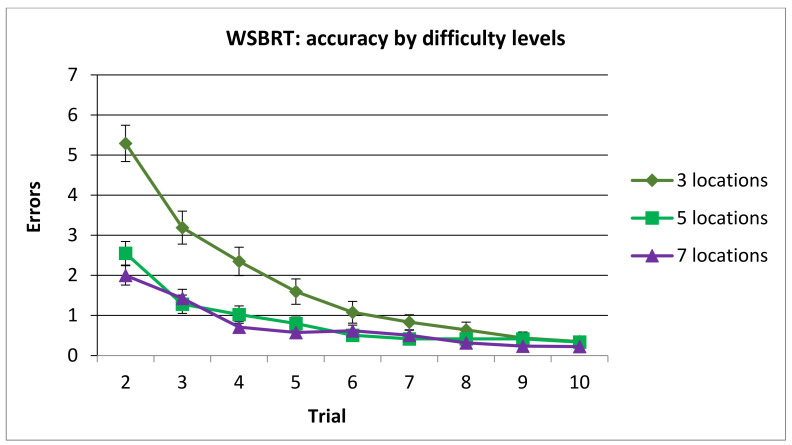
Mean number of errors committed in the WSBRT according to the difficulty. Note that trial 1 was removed from analysis since performance was at random. (Mean ± SEM).

In addition, the number of errors in 3 locations’ condition was higher in trials 2, 3, 4 and 5 compared with 5 and 7 locations’ conditions. Analyses also revealed that the number of errors in trial 2 was higher in 5 locations’ condition compared with 7 locations’ condition (see Figure 5).

#### 3.1.2. Latency

ANOVA (Sex × Difficulty × Trial) revealed a significant main effect of Sex F(1, 87) = 25.45, *p* < 0.000, η_2_ = 0.226; and Trial F(8, 696) = 111.46, *p* < 0.000, η_2_ = 0.561 but not in Difficulty F(2, 174) = 0.27, *p* = 0.757. Significant differences were also observed in the interaction terms Sex × Trial, F(8, 696) = 2.1, *p* = 0.033, η_2_ = 0.023 and Difficulty × Trial F(16, 1392)= 9.64, *p* < 0.000, η_2_ = 0.099.

**Figure 6:**
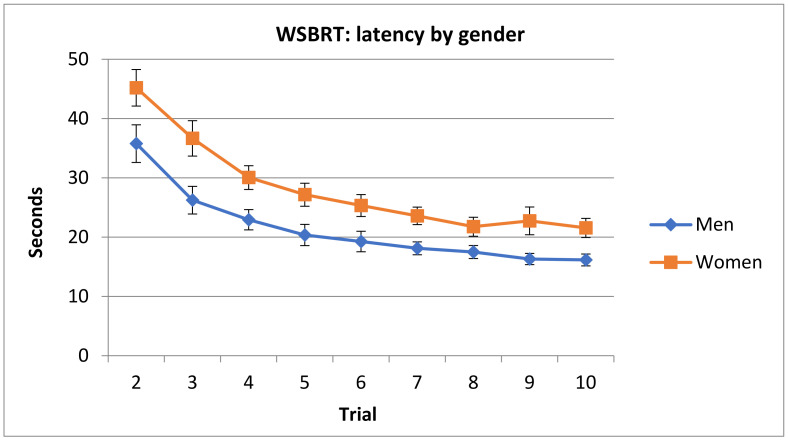
Time spent by men and women in the WSBRT. Note that trial 1 was removed from analysis since performance was at random. (Mean ± SEM).

Post hoc analysis of Sex x Trial revealed that both men and women reached the asymptotic level in trial 6. In addition, men spent less time completing all trials than women, except in trials 7 and 8 (see Figure 6).

Post hoc analysis of Difficulty x Trial showed that the asymptotic level was reached in trials 7, 5 and 4, for 3, 5 and 7 positions’ conditions, respectively.

**Figure 7:**
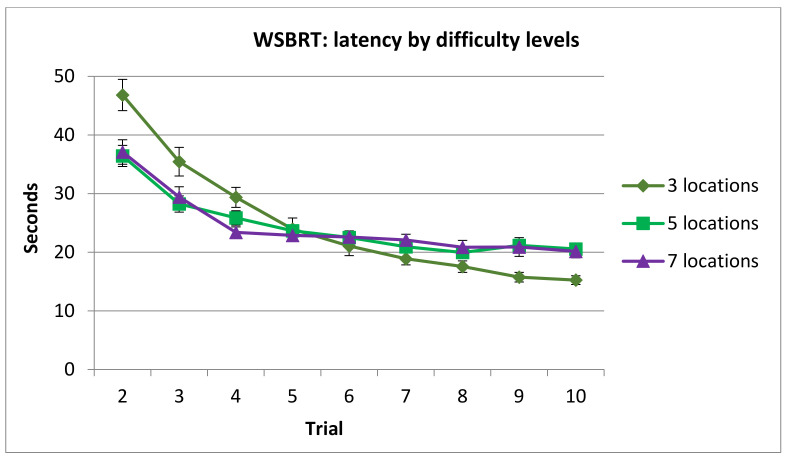
Time spent completing each trial of each difficulty condition in the WSBRT. Note that trial 1 was removed from analysis since performance was at random. (Mean ± SEM).

Moreover, time spent in 3 locations’ condition was longer compared with 5 locations’ condition in trials 2, 3 and 4, but the opposite occurred in trials 9 and 10 (see Figure 7).

### 3.2. NWSBRT

#### 3.2.1. Number of Errors

ANOVA (Sex × Difficulty × Trial) revealed significant differences in Difficulty F(2, 188)= 38.05, *p* < 0.000, η_2_ = 0.288 and Trial F(8, 752)= 47.33, *p* < 0.000, η_2_ = 0.334 but not in Sex F(1, 94)= 2.6, *p*= 0.109. In addition, there was a significant main effect of the interaction term Difficulty × Trial F(16, 1504)= 3.75, *p* < 0.000, η_2_ = 0.038, but not in other interactions: Difficulty × Sex F(2, 188)= 0.03, *p* = 0.97; or Trial × Sex F(8, 752) = 1.5, *p* = 0.151.

Post hoc analysis of Difficulty x Trial interaction revealed that the asymptotic level was reached in trial 6 in the 3, 5 boxes’ conditions and in trial 5 in the 7 boxes’ conditions, respectively.

**Figure 8:**
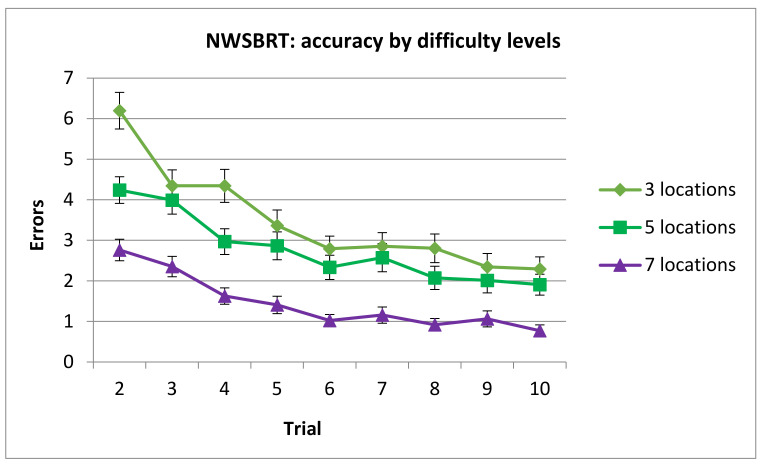
Mean number of errors by difficulty levels and trials in the NWSBRT. Note that trial 1 was removed from analysis since performance was at random. (Mean ± SEM).

In addition, number of errors in trials 2 and 4 was higher in 3 locations’ condition compared to 5 locations’ condition. The number of errors in all trials of 3 locations’ condition was higher compared to 7 locations’ condition and number of errors in all trials of 5 locations’ condition was higher compared to 7 locations’ condition (see Figure 8).

#### 3.2.2. Latency

ANOVA (Sex × Difficulty × Trial) showed significant main effect of Difficulty F(2, 188) = 8.08, *p* < 0.001, η_2_ = 0.79 and Trial factors F(8, 752) = 53.65, *p* < 0.000, η_2_ = 0.363, but not in Sex F(1, 94) = 1.96, *p* = 0.164. Significant results were found in Difficulty × Trial interaction term F(16, 1504) = 3.77, *p* < 0.000, η_2_ = 0.038 but not in Difficulty × Sex F(2, 188) = 0.268, *p* = 0.764 or Trial × Sex F(8, 752) = 1.071, *p* = 0.38.

Post hoc analysis of the interaction term Difficulty × Trial revealed that the asymptotic level was reached in trials 5, 6 and 5 for 3, 5, and 7 locations, respectively.

**Figure 9:**
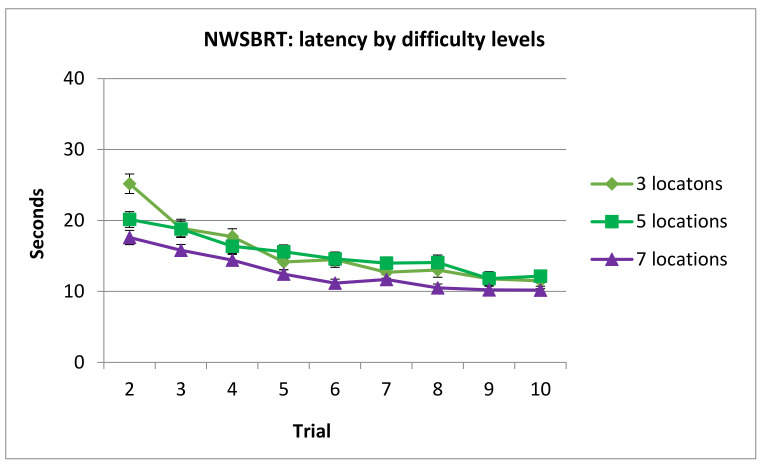
Time spent in NSBRT by trials and difficulty levels. Note that trial 1 was removed from analysis since performance was at random. (Mean ± SEM).

On the other hand, participants spent more time in trial 2 in 3 locations’ condition than in the same trial in the 5 locations’ condition. Finally, time spent in trials 2, 3, 4 and 6 in 3 locations’ condition was longer compared to 7 locations’ condition and latencies in trials 2, 4, 5 and 7 in 5 locations’ condition were longer compared to 7 locations’ condition (see Figure 9).

### 3.3. ASMRT

#### 3.3.1. Number of Correct Answers

ANOVA (Sex × Difficulty) revealed significant differences in both factors: Sex F(1, 100) = 8.35, *p* = 0.004, η_2_ = 0.077; Difficulty F(2, 200) = 4.23, *p*= 0.015, η_2_ = 0.04, and their interaction Difficulty × Sex F(2, 200) = 3.76, *p* = 0.025, η_2_ = 0.036.

**Figure 10:**
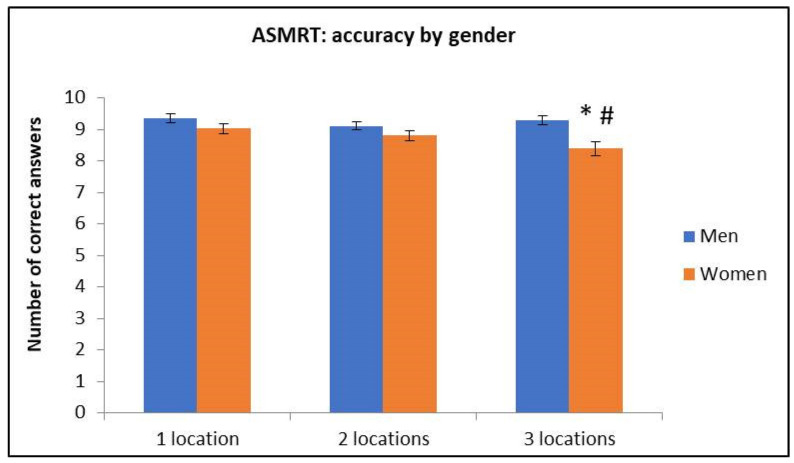
Number of correct answers in the ASMRT by Sex and Difficulty. * Differences between women and men; # Differences with 1-2 locations in women. (Mean ± SEM).

Post hoc analysis of Sex x Difficulty interaction revealed that women committed more errors in 3 locations’ condition compared to all conditions in men. In addition, they also committed more errors in 3 locations’ condition compared to 1 and 2 locations’ conditions (see Figure 10).

#### 3.3.2. Reaction Time

ANOVA (Sex x Difficulty) showed significant differences in Sex F(1, 100) = 11.16, *p* = 0.001, η_2_ = 0.1 but neither in Difficulty F(2, 200)= 0.86, *p* = 0.424 nor in the interaction Difficulty × Sex F(2, 200)= 0.9, *p* = 0.404.

Analyses revealed that men were faster than women (1850 vs. 2273 milliseconds).

## 4. Discussion

The aim of this work was to describe the spatial behavior of men and women in three spatial memory tasks with slightly different demands. Specifically, we tested how different factors, such as familiarity, difficulty and the active or passive role of the participant could influence spatial behavior in men and women. Results showed differences according to the difficulty level in the three tests. These differences were smaller in the ASMRT. The active/passive role of the participants, familiarity with the environment and the amount of spatial information provided in each trial are factors that could explain these differences. Regarding sex, significant differences were found in the WSBRT and the ASMRT but not in the NWSBRT. In this case, the cognitive load required to perform the task, the spatial strategy used and the familiarity effect are the factors that could explain the differences found between men and women.

The WSBRT and the NWSBRT have the same design (remembering 3, 5 and 7 boxes out of a total of 16); nevertheless, accuracy at different levels of difficulty could indicate that NWSBRT is more demanding than WSBRT, as has been shown before [56]. According to our results, differences between the three levels of difficulty were present in a higher number of trials in the NWSBRT as compared to WSBRT. What is more, the asymptotic level could also provide information about task difficulty. Thus, participants reached this optimum level of performance sooner in the WSBRT.

Note that NWSBRT allows a general vision of the room in a single glance; however, participants cannot walk inside the virtual room. Due to the impossibility of freely navigating, participants cannot establish as many relationships between rewarded boxes and available landmarks in the room as in the WSBRT. This situation forces participants to use an allocentric reference frame to remember rewarded positions (a reward box could be related to any stimuli of the room). Conversely, since participants can move around and explore inside the virtual room in the WSBRT task, rewarded positions could be achieved using different strategies using more than one reference frame.

Furthermore, familiarity with the task and context affects performance in WSBRT and NWSBRT. Thus, despite the increasing number of boxes to memorize in 5 and 7 boxes’ conditions, accuracy improved in comparison to 3 rewards’ condition. Number of errors and latencies supported this statement. Thus, longer latencies were observed in 3 locations’ condition than in 5 and 7 rewards’ conditions. These results could indicate that previous experience with the environment and procedural learning could improve performance in other more demanding conditions (5 and 7 rewards). This effect of familiarity was also reported in other tasks [33,57].

On the other hand, the ASMRT has a smaller total number of boxes and fewer locations to memorize compared with the WSBRT and NWSBRT: 1, 2 or 3 locations to memorize out of a total of 9 positions. In addition, ASMRT combines the first-person view of WSBRT and the passive contact with the environment of the NWSBRT. These conditions forced participants to memorize the spatial position with a reduced vision of the room; that is, incomplete spatial information makes the creation of a complete reference frame of the room difficult. Participants are forced to imagine the whole configuration of the room, the location of the landmarks and the disposition of the boxes through the union of the different screenshots. As they complete the test, more spatial information about the room is gathered to create a cognitive map to localize the rewarded boxes. These data agree with previous research with the ASMRT, where correlations between the different levels of difficulty and the visuospatial span measured with the Corsi Block Tapping Test were found [58].

Therefore, the ASMRT requires having a large visuospatial working memory, an area where women are generally less skillful [35,59]. Results obtained in the ASMRT could be reflecting once again the difficulties of women in performing allocentric tasks with high demands on visuospatial working memory [33,35,60,61].

In line with the above, it is well known that men and women have different preferences when choosing spatial strategies. Men have a predisposition to attend to spatial information related to the shape of the scenery, distances and Euclidean information, whereas women tend to focus on the landmarks available in the environment. As a result, men generally use allocentric reference frames in a flexible way, which allows them to be oriented from different locations of the scenery. On the other hand, women tend to create routes from one point to another and rely heavily on information related to the available landmarks to guide themselves [41,62,63]. The spatial strategies used by men are more adaptive to solving allocentric spatial tasks, creating cognitive maps and recognizing spatial environments independently of the point of view [54,62]. Results obtained in both the ASMRT and the WSBRT confirm this. The continual change of point of view forces participants to use allocentric strategies and create a mental configuration of the room to orient themselves and calculate the position of the rewarded boxes. The apparent slowness of women reflected in reaction times and latencies data can be explained by their predisposition to the use of certain spatial strategies, which are not always the most appropriate [41]. Indeed, sex differences in topographic memory are represented in the time of exposure to the environment and the number of repetitions required to learn (see [33]). Generally, women need more time to learn and more repetitions than men.

However, sex dimorphism can disappear under very high or very low difficulty conditions. An example of this is the NWSBRT, where no sex differences were found, probably due to its high cognitive demand, as mentioned above. Another example is the work of Cánovas and colleagues [34], who applied the three difficulty levels of the WSBRT to different groups so that there was no possibility of improving between levels of difficulty. Sex dimorphism only appeared under the medium level of difficulty and not in the easiest or the most difficult conditions. In the current investigation, all participants completed the three difficulty conditions and showed sex dimorphism regardless of the difficulty level. So, they had a previous experience with the environment to face the last and most difficult level.

Moreover, familiarity with the task has been reported to affect appearance of sex differences. Hence, Nori and colleagues [31] confirmed that familiarity reduces or eliminates sex differences in spatial memory tasks. Furthermore, women can be as good as men at carrying out survey-type tasks in highly familiar environments [51,61]. In addition, it was reported that participants tend to use egocentric strategies in new contexts, whereas they are more prone to using allocentric orientation in familiar environments [64]. With this in mind, the possibility could be considered that this would also happen in our study as well. That is, during the first trials, participants could try to solve the task using egocentric strategies, but some trials later, when they were more experienced with the context, they could switch to an allocentric strategy. It was demonstrated that men and women need different lengths of time to achieve the same precision in spatial tasks [33,37,41], which is reflected in the asymptotic level data of our tests. Therefore, this delay in women in reaching the asymptotic execution may be due to two reasons: first, because the change between strategies occurs later; second, because they have a preference for using spatial reference frames more related to egocentric strategies [41,62,63]. Once the spatial context is well known, men and women are able to work with it equally, increasing their capability to solve these spatial problems [5,33,63].

## 5. Conclusions

In conclusion, this research has the novelty of studying the differences in spatial memory abilities in men and women using three assessment tools with slightly different demands, which allows a more detailed analysis of the factors that determine sex dimorphism. Specifically, it was found that performance in these different spatial tasks is determined by the difficulty level, the familiarity with the spatial context, the possibility of navigating within the scenery and the amount of visual information given in each trial. All these factors are related to the ability to use allocentric reference frames, accuracy in creating cognitive maps, the ability to perform mental rotations and visuospatial span capacity. In all these abilities men are generally more skillful [35,62,65], which is reflected in the results obtained in the three spatial tasks. These tests highlight the sexual differences in the methods of processing and understanding the space. Since some studies report that men are more experienced than women with virtual reality worlds and this could affect their performance in virtual reality tasks [66,67], for future research it would be interesting to add information about videogames experience that could enhance the conclusions reached.

## Figures and Tables

**Table 1:** Distribution of the sample according to the spatial memory test applied.

	Men	Women	Total
**WSBRT**	n = 48 (47% Spanish) Age = 22.85 ± 2.89	n = 41 (56% Spanish) Age = 20.56 ± 2.17	n = 89 (51% Spanish) Age = 21.8 ± 2.82
**ASMRT**	n = 64 (31% Spanish) Age = 22.67 ± 3.34	n = 38 (44% Spanish) Age = 21.1 ± 2.6	n = 102 (36% Spanish) Age = 22.17 ± 3.19
**NWSBRT**	n = 46 (39% Spanish) Age = 22.42 ± 2.49	n = 50 (40% Spanish) Age = 21.62 ± 2.82	n = 96 (39% Spanish) Age = 22.01 ± 2.68
			n = 287 (42% Spanish) Age = 22 ± 2.9

**Table 2:** Scheme of the three spatial tests.

	WSBRT	ASMRT	NWSBRT
**Total number of boxes**	16	9	16
**Locations to memorize**	3, 5 and 7	1, 2 and 3	3, 5 and 7
**Trials of each difficulty level**	10 (9 analyzed)	4	10 (9 analyzed)
**View**	First-person view	First-person view	Survey perspective
**Participant’s interaction with the context**	Active	Passive	Passive

## Data Availability

Not applicable.
